# Auditory Neuropathy / Auditory Dyssynchrony in children with Cochlear Implants

**DOI:** 10.1590/S1808-86942011000400012

**Published:** 2015-10-19

**Authors:** Ana Claudia Martinho de Carvalho, Maria Cecilia Bevilacqua, Koichi Sameshima, Orozimbo Alves Costa Filho

**Affiliations:** 1 Doctoral degree, speech therapy at the Núcleo do Ouvido Biônico (Bionic Ear Center), São Paulo Samaritan Hospital; 2 Full professor, São Paulo University; 3 Associate professor, São Paulo University; 4 Full professor, São Paulo University

**Keywords:** cochlear implantation, auditory perception, Rehabilitation of Hearing Impaired

## Abstract

**Abstract:**

The electrical stimulation generated by the Cochlear Implant (CI) may improve the neural synchrony and hence contribute to the development of auditory skills in patients with Auditory Neuropathy / Auditory Dyssynchrony (AN/AD).

**Aim:**

Prospective cohort cross-sectional study to evaluate the auditory performance and the characteristics of the electrically evoked compound action potential (ECAP) in 18 children with AN/AD and cochlear implants.

**Material and methods:**

The auditory perception was evaluated by sound field thresholds and speech perception tests. To evaluate ECAP's characteristics, the threshold and amplitude of neural response were evaluated at 80Hz and 35Hz.

**Results:**

No significant statistical difference was found concerning the development of auditory skills. The ECAP's characteristics differences at 80 and 35Hz stimulation rate were also not statistically significant.

**Conclusion:**

The CI was seen as an efficient resource to develop auditory skills in 94% of the AN/ AD patients studied. The auditory perception benefits and the possibility to measure ECAP showed that the electrical stimulation could compensate for the neural dyssynchrony caused by the AN/AD. However, a unique clinical procedure cannot be proposed at this point. Therefore, a careful and complete evaluation of each AN/AD patient before recommending a Cochlear Implant is advised. *Clinical Trials*: NCT01023932

## INTRODUCTION

The approach for habilitating and rehabilitating patients with auditory neuropathy/auditory dys-synchrony (AN/AD) has raised diverging questions and opinions, because these individuals continue to be placed in the special cases group for cochlear implants. The term auditory neuropathy has been used generically to name discrepancies between the cochlear and neural functions of the auditory system.

AN/AD has been described in the literature as a change in neural synchronism whereby the function of outer hair cells is preserved while afferent neural transmission is compromised. However, to this date the exact injury site has not been demonstrated by current methods in clinical practice.

AN/AD can significantly affect speech understanding and production. Thus, adequate habilitation and rehabilitation is needed especially in children, who go through critical hearing and language development periods.

Individual hearing aids have shown poor results; amplification may improve the detection of sounds but does not provide any benefit for the development of speech discrimination, recognition, and understanding.^1^, ^2^, ^3^

Other communication strategies - communication by gestures, lip reading, and frequency modulated (FM) systems - have been suggested for habilitation and rehabilitation in this group of patients^4,^^5^.

About one third of AN/AD patients present severe to profound hearing loss, and are thus candidates for cochlear implants^6^.

Cochlear implants are indicated in this group because this electronic device can partially replace the function of auditory sensory cells. These devices stimulate the auditory nerve directly, thereby improving neural synchrony and supporting language and hearing development^7^, ^8^, ^9^.

At the same time, the compound action potential of the electrically evoked auditory nerve (ECAP) or neural response in cochlear implant users can objectively demonstrate changes in hearing following electrical stimulation of the auditory system in AN/AD patients.

Because the electrical stimulation of cochlear implants may improve neural synchrony and support auditory ability development, the purpose of this study was to assess hearing performance and ECAP features in a group of AN/AD children with cochlear implants.

## MATERIAL AND METHODS

A cross-sectional cohort study was undertaken of 18 AN/AD children with cochlear implants for at least six months that participated in two different cochlear implant programs in São Paulo state.

The institutional ethics review board for research in human being approved this study (no. 316/2005 UEP-CEP).

Parents and caretakers of participants were informed about the procedures, and signed a free informed consent form.

Regarding the inclusion criteria, the surgical indication for cochlear implants in children with prelingual hearing loss is made in both centers according to international criteria, namely: age around 1 year; cochlear permeability for inserting the electrodes surgically; bilateral severe to profound and/or profound sensorineural hearing loss; auditory threshold with normal amplification over 60 dB at 500, 1,000, and 2,000 Hz after intense and effective auditory habilitation; limited benefit for auditory abilities with the use of hearing aids; absence of intellectual or emotional deficits; motivated family for using cochlear implants and to develop favorable communication attitudes in the children; adequate family expectation about the results of cochlear implants; participation of children in auditory habilitation and rehabilitation programs in their city of origin.

Preoperative imaging exams (magnetic resonance imaging and computed tomography of the temporal bones) were normal in all subjects.

Other clinical peculiarities not mentioned above were analyzed in each patient and defined as special cases.

Cochlear implants are not indicated in prelingual hearing impaired children also according to international criteria in both cochlear implant programs from which data were gathered, as follows: severe neurological conditions associated with hearing loss; medical or psychological conditions that contraindicate surgery; hearing loss due to cochlear or auditory nerve agenesis, or central injuries; active middle ear infection; unreal family expectations about the benefits, results, and limits of cochlear implants.

The audiological and electrophysiological features of AN/AD in the study sample were as follows: normally functioning OHCs identified by otoacoustic emissions (OAE) and/or cochlear microphonism; neural alterations in which brainstem auditory evoked potentials were markedly altered or absent; altered audiometric thresholds; limited results with conventional amplification in hearing and language development.

The age at which surgery was done in the study sample ranged from 1 year and 8 months to 6 years and 11 months (mean - 3 years and 8 months). The duration of cochlear implant use ranged from 10 months to 3 years and 5 months.

The cochlear implant brand and model in the study subjects were not taken into account in the inclusion/ exclusion criteria. Nevertheless, all subjects used the Nucleus 24 cochlear implant (Cochlear Corporation) and the electrodes were completely inserted.

Based on Davis and Silverman's (1970)^10^ hearing loss classification, 9 subjects (50%) had profound hearing loss, and 9 subjects (50%) had severe hearing loss as evidenced by their audiometric thresholds.

Data were taken from files of prenatal, perinatal, and postnatal registries, the etiology of hearing loss, audiologic and electrophysiologic tests (pure tone audiometry, OAE, impedance testing, BAEP, and presence of cochlear microphonism), and the cochlear implant surgery.

Behavioral assessment of hearing (play audiometry to measure the free field audiometry threshold at 500 Hz, 1 kHz, 2 kHz, and 4 kHz while using cochlear implants) and speech perception assessment (test adapted to age and hearing abilities, adapted into Portuguese for each age level) were used to test hearing perception.

The following procedures were used for testing speech perception: procedure to assess children with profound hearing loss^11^ adapted from the Glendonald Auditory Screening Procedure (GASP), and perception of speech sounds in children - word list^12^.

The nature of hearing ability tasks in study subjects was classified according to the observed hearing abilities in the speech perception assessment procedures.

The category “detection of sounds” was used in children with results showing limited speech perception that were able to carry out only Test 1 of the GASP.

The closed set category was done in children that were able to carry out Test 5 of the GASP, in which children are presented with alternative answers^13^.

Children that were able to carry out Test 6 of the GASP - and therefore the list of disyllables - were classified in the open set category. In this test, children had no choice of answers, and required more complex hearing abilities^13^.

Impedance telemetry or reverse telemetry or bidirectional telemetry was done in all stimulation modes present in the Nucleus 24 cochlear implant system to assess the neural response; the aim was to analyze the integrity of the intracochlear electrodes.

The impedance values of intracochlear electrodes 20, 15, 10, and 5 were evaluated.

Electrodes with altered impedance values - indicating a short circuit or open circuit - were excluded from the evaluation. The adjacent apical electrode (that had normal impedance values) was then used in the research protocol.

Four out of 72 electrodes had altered impedance values; therefore, adjacent apical electrodes were used.

The ECAP was carried out with electrodes 20, 15, 10 and 5, which were in the surgically inserted electrodes.

The parameters that were applied in recording the neural response were taken from published recommendations^14^, ^15^, ^16^.

ECAP threshold measurements were taken at the 80 and 35Hz stimulation frequencies to assess possible changes in the characteristics of the neural response as the stimulation frequency is reduced. The same neural response recording parameters were used for the two tested stimulation frequencies. ECAP responses were classified according to the following parameters: presence of N1 (measurable peak); presence of P1/P2 (measurable peak); response morphology; response reproducibility; and consecutive valid recordings.

Neural response amplitude and threshold value criteria were applied to assess the recordings qualitatively and quantitatively.

Two-way analysis of variance was applied to evaluate correlations between age at surgery, duration of cochlear implant use, and speech perception results.

Fisher's exact test was applied to check the relation between TOAE results and hearing abilities.

Repeated measures analysis of variance was applied to study the relationship between mean speech frequency auditory thresholds (0.5, 1 and 2 kHz), and open and closed set speech recognition results in each subject. Free field auditory threshold means at each frequency were marked with triangles at different tested frequency ranges.

Repeated measures analysis of variances was also applied to analyze neural response amplitude and thresholds at 35 and 80 Hz.

## RESULTS

The causes of hearing loss in the study sample were as follows: association of multiple indicators of hearing loss at birth (or multifactorial causes) in 50% of subjects; unknown or idiopathic in 44% of subjects; and congenital rubella in 6% of subjects.

Speech perception results (speech detection, closed set auditory recognition, and open set auditory recognition) were distributed in the study population as follows: closed set speech recognition - 61% of subjects; open set speech recognition - 33% of subjects; and detection of sounds only in 6% of subjects.

There were no statistically significant differences among the mean ages at surgery, the duration of use of cochlear implants, and speech recognition auditory abilities.

There was no statistically significant relationship in speech perception performance for open and closed set speech recognition and the presence of OAE before surgery in the right ear (*p*=0.304) and left ear (*p*=0.620).

**Figure 1:**
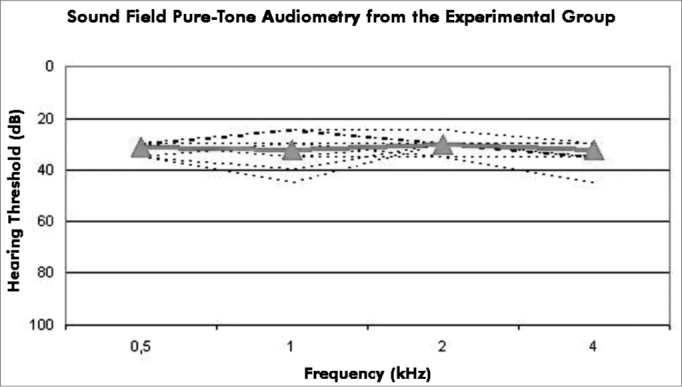
Free Field Pure Tone Audiometry - Results of free field auditory thresholds (dB) at 0.5, 1, 2, and 4 kHz.

Figure 1 shows the results of free field auditory thresholds at 0.5, 1, 2 and 4 kHz in each study subject.

There were no statistically significant differences among the means of pure tone thresholds at speech frequencies or in open and closed set speech recognition abilities (*p*=0.485).

Neural response recordings were: 83% of measurements in electrode 20, followed by 83% of positive response in electrode 15, then 88% in electrode 10, and 94% of recordings in electrode 5.

**Figure 2:**
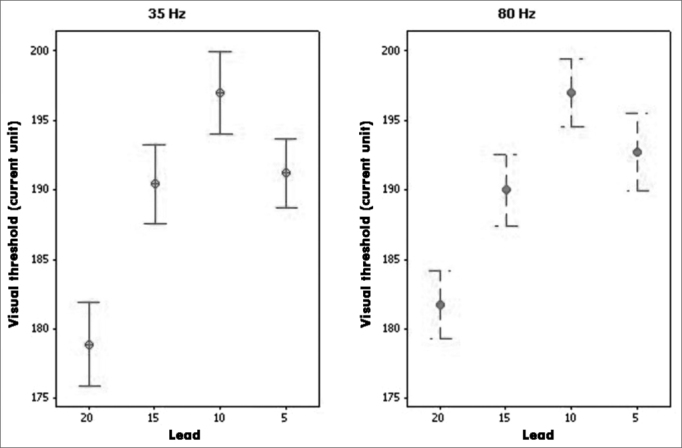
Visual Neural Response Thresholds - Results of means ± standard errors of the visual neural response threshold in electrodes 20, 15, 10, and 5, at 35 and 80 Hz stimulation frequencies.

Figure 2 shows a comparison of neural response visual thresholds at 35 and 80 Hz (stimulus frequencies), as observe in electrodes 20,15, 10 and 5.

A difference was found in the threshold means of four electrodes (*p*=0.000) at both 35 Hz and 80 Hz stimulus frequencies.

Specific analysis per electrode revealed no differences in visual threshold means at 35 and 80 Hz stimulus frequencies (*p*=0.566).

The results of comparing electrodes revealed that the visual threshold mean was lower in electrode 20 compared to the other electrodes (*p*=0.000). The visual mean of electrode 15 was lower than that of electrode 10. A comparison of the visual threshold means in electrodes 10 and 5 yielded a marginal p value (*p*=0.051).

The neural response amplitude ranged from 21.8 mV to 128 mV in electrodes 20, 15, 10, and 5 at a stimulus frequency of 35 Hz. The neural response amplitude varied from 27.7 mV to 103.1 mV at a stimulus frequency of 80 Hz among the tested electrodes.

**Figure 3:**
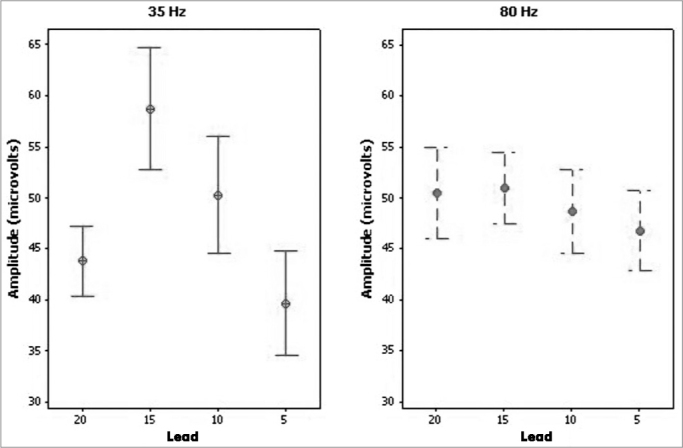
Neural Response Amplitude - Results of means ± standard errors of the neural response amplitude in electrodes 20, 15, 10, and 5, at 35 and 80 Hz stimulation frequencies.

Figure 3 shows the mean neural response amplitude values in electrodes 20, 15, 10, and 5 at 35 and 80 Hz stimulus frequency.

There were no statistically significant differences among the neural response amplitude means at stimulus frequencies of 35 and 80 Hz (*p*=0.969) in all tested electrodes.

A comparison of neural response amplitudes among electrodes revealed no statistically significant differences among the neural response amplitude means in electrodes 20,15, 10, and 5 (*p*=0.096) at stimulus frequencies of 35Hz and 80Hz (*p*=0.527).

## DISCUSSION

The variable results attained in users of cochlear implants is closely related with intrinsic factors pertaining to the hearing system of each individual and extrinsic factors such as motivation for using the device, family support, habilitation and rehabilitation method, among others.

In the AN/AD population, these widely diverging results may reflect degrees of disease or changes along auditory pathways.

Secondary AN/AD to neonatal diseases was encountered in 50% of the study subjects. Complications during and/or after birth as related factors in the diagnosis of this disease have been described in the literature^17^, ^18^, ^19^, ^20^, ^21^, ^22^.

The difficulties for establishing the etiology of AN/ AD are evident in this study - 44% of subjects had AN/ AD of unknown causes. A previous study^22^ also reported a similar rate: 40% of 70 patients had idiopathic AN/AD.

Congenital rubella as a cause of AN/AD has not been described in the literature, possibly because this disease has been controlled in developed countries by immunization of women of reproductive age.

We found encouraging results of using electrical stimulation in AN/AD patients; hearing abilities improved significantly in 94% of our study subjects. Promising results of cochlear implant use in AN/AD patients have already been published^7,^^20,^^23,^^24^.

Thus, cochlear implants appeared to help develop auditory abilities irrespective of a diagnosis of AN/AD. This finding concurs with those of other published studies^7,^^23^.

Speech perception results were better the longer cochlear implants were used, even though there were no statistically significant differences in duration of cochlear implant use and open and closed set speech recognition. Other authors have also pointed out a gradual development of speech perception abilities following cochlear implants use for some time in different groups of children^25^, ^26^, ^27^, ^28^, ^29^, ^30^, ^31^, ^32^, ^33^.

The mechanism by which OAE deteriorate or are lost in AN/AD patients remains unknown; no significant relationship between absence and/or possible loss of OAE and any specific changes in AN/AD were found. Our results showed that the presence of OAE preoperatively did not seem to be a determining variable for the development of hearing abilities, given that no significant correlation was found between the presence of OAE in right and left ears and open and closed set speech recognition abilities.

Thus, the presence of OAE preoperatively did not necessarily mean a better prognosis after placing cochlear implants. Lack of a significant correlation between the presence of OAE and speech perception results in hearing aid users has already been published in the literature^34^.

All study subjects had typical mean audiometry threshold values for users of cochlear implants. Minor variations may occur because audiometry threshold measurements are subjective - attention, concentration, and motivation during the two or more test situations may result in small differences.

A few studies have reported the neural response recording features of ECAP in AN/AD patients; our study sample had no specific aspects related to this potential.

No specific features were found in the ECAP in the study sample (children with AN/AD). This condition, therefore, did not appear to be a determining factor for ECAP recordings.

Our analysis was based on existing studies on ECAP in cochlear implant users in general because of the similarity between ECAP findings in our study sample and in the clinical population of cochlear implants users, and because there have been few studies describing specifically the neural response characteristics in AN/AD patients.

Neural responses were present in over 80% of our recordings. Similar possibilities in ECAP recordings have been described elsewhere^35^.

Charasse et al. (2004)^36^ have suggested that the stimulus frequency for recording ECAP can affect the number of valid recording and the neural response quality in cochlear implant users.

Using lower stimulus frequencies in AN/AD cases could presumably generate more robust neural responses; thus, altered neural synchrony could be the cause of speech perception difficulties.

The hypothesis that the hearing system responds more slowly in AN/AD patients because of altered auditory nerve neuron triggering, and that the resulting lower stimulus presentation rate generates more evident neural responses, motivated us to measure the neural response characteristics at stimulus frequencies of 35 and 80 Hz.

However, no statistically significant differences between visual neural response thresholds were found when comparing the two stimulus frequencies. These findings corroborate previous papers^35^, in which no significant differences in ECAP recordings at 35 Hz and 80 Hz were found in adult cochlear implant users.

Similarly, there were no statistically significant differences in neural response amplitudes as the stimulus frequency was reduced to 35 Hz. However, the mean neural response amplitude values were higher at 35 Hz in all electrodes, indicating better neural response measurements at this stimulus frequency. Other authors have also reported that neural response amplitudes tended to increase at lower stimulus frequencies^37^.

Similarities in neural response characteristic at stimulus frequencies of 35 and 80 Hz suggests that the auditory system in the study population retrieved the temporal properties in coded information, following electrical stimulation, irrespective of the stimulus frequency. Significant differences in the quality of neural responses have been described in the literature only for recordings obtained at stimulus frequencies over 150 Hz^36^. Further studies investigating neural response characteristics in AN/AD patients at stimulus frequencies over 80 Hz may contribute to the analysis of neural response recording quality as stimulus frequencies are raised significantly to values over 150 Hz.

In the present study, the benefits for hearing perception seen in AN/AD children that use cochlear implants - and the possibility of recording ECAP - have shown that electric stimulation from cochlear implants compensated for the altered neural synchrony caused by AN/AD.

Except for one study subject in our series, cochlear implant use in AN/AD children resulted in comparable development of hearing abilities to children without AN/ AD that also used cochlear implants. Intrinsic variables that are inherent to the pathophysiology of AN/AD did not appear to affect the development of hearing abilities in AN/AD patients following electric stimulation. AN/AD children do not require specific adjustments of programming parameters for the speech processor.

It should be noted that the results of cochlear implant use in AN/AD patients were closely related with the site of the alteration. However, it is sill not possible to define exactly the injury site to specify which AN/AD cases could benefit most from cochlear implants. Encouraging results in our study sample related to the development of hearing abilities in AN/AD patients using cochlear implants suggest that neural function is preserved. Altered neural synchrony in these cases was possibly due to altered outer hair cells and/or the synapses of these cells with the auditory nerve.

A longitudinal study of AN/AD patients that use cochlear implants may clarify specific nuances in the rehabilitation process of this group as these patients use cochlear implants. At the same time, a test protocol that includes an assessment of hearing abilities in the presence of competing noise, of language abilities, and of speech production, may help establish the specific clinical features of AN/AD patients that use cochlear implants.

## CONCLUSION

In this study, cochlear implants are effective measures for developing auditory abilities in 94% of AN/AD subjects. Use of a reduced stimulation frequency (35 Hz) did not result in statistically significant changes in neural response amplitudes and thresholds.

However, a common approach cannot yet be defined and adopted, given the heterogeneous nature of our clinical group of cases.
